# Assessing real-world natural history of indolent systemic mastocytosis: A retrospective matched cohort study

**DOI:** 10.1016/j.jacig.2026.100701

**Published:** 2026-04-13

**Authors:** Thanai Pongdee, Dakota Powell, Taylor Weis, Robyn Scherber, Teresa Green, Allison Smither, Purushotham Krishnappa, Katherine Carlson, Prerna Singh, Steve Duff, Erin Sullivan

**Affiliations:** a Division of Allergic Diseases, Mayo Clinic, Rochester, Minn; b Blueprint Medicines Corporation, Cambridge, Mass; c Department of Hematology/Oncology, University of Texas Health San Antonio, San Antonio, Tex; d nference, Inc, Cambridge, Mass; e nference, Inc, Bengaluru, India; f Veritas Health Economics Consulting, Kamas, Utah

**Keywords:** Indolent systemic mastocytosis, electronic health records, symptom burden, health care resource utilization, KIT D816V mutation, natural language processing, real-world disease burden, comorbidity burden

## Abstract

**Background:**

Indolent systemic mastocytosis (ISM) is the most common form of systemic mastocytosis, accounting for more than 80% of cases. Patients with ISM experience severe, unpredictable symptoms, including potentially life-threatening anaphylaxis. As a chronic condition, understanding its longitudinal natural history is essential.

**Objective:**

We sought to evaluate the real-world disease burden and describe the natural history of ISM by comparing patients treated at Mayo Clinic to a matched control cohort. Innovative natural language processing methods were used alongside traditional structured data analysis.

**Methods:**

A retrospective cohort study was conducted using data from patients diagnosed with ISM at Mayo Clinic between January 1, 2005, and June 30, 2022. Patients were identified using structured electronic health record data and natural language processing of clinical notes. A control cohort was created using 10:1 propensity matching based on demographic and clinical variables. Baseline characteristics, symptoms, clinical outcomes, and health care utilization were assessed.

**Results:**

The study included 203 patients with ISM and 2030 matched controls. Patients with ISM had a higher symptom burden (mean, 7.4 vs 4.8 at baseline) and significantly more comorbidities. Bone involvement (osteopenia/osteoporosis) was present in 68% of patients with ISM versus 28% of controls (*P* < .0001). Patients with ISM had higher health care utilization and medication use.

**Conclusions:**

Patients with ISM experience a high burden of heterogeneous symptoms and elevated rates of bone disease. These findings support the consideration of disease-modifying therapies in ISM management.

Systemic mastocytosis (SM) is a clonal mast cell disease with advanced and nonadvanced variants, both of which are primarily driven by the *KIT* D816V mutation in approximately 95% of cases.^1^ Indolent systemic mastocytosis (ISM) is the most common SM subtype, accounting for more than 80% of all SM cases,^2^^,^^3^ with a prevalence of approximately 1 in 5000.^2^^,^^4^, ^5^, ^6^

Although unlikely to significantly impact life expectancy like the advanced form of SM, patients with ISM experience severe and unpredictable symptoms including potentially life-threatening anaphylaxis.^7^^,^^8^ In addition, patients often suffer with debilitating skin, gastrointestinal, musculoskeletal, and systemic symptoms. Many patients may experience symptoms across all these domains and, absent adequate disease control, these symptoms can worsen over time and result in patients developing comorbid conditions, such as osteoporosis or osteopenia.^9^, ^10^, ^11^, ^12^, ^13^, ^14^, ^15^, ^16^ In recent patient surveys, patients have reported significant impact on quality of life, mood, and ability to work as a result of disease.^17^ These symptoms cause reduced patient quality of life, including impairment of daily activities, mood, and ability to work, and increased use of health care resources.^7^^,^^8^

Although several treatment options now exist for treating SM (midostaurin and avapritinib for advanced SM and avapritinib for ISM), clinical trials of these therapies are not designed to elucidate long-term disease burden, real-world practice patterns, or a patient’s diagnostic journey. These issues are best explored, especially for rare diseases, with the use of large, longitudinal electronic health record (EHR) databases, often with millions of patient records.^18^^,^^19^ Technological advancements in artificial intelligence, natural language processing (NLP), and machine learning can now amplify researchers’ analytic capabilities with these data sources.

The objectives of this study were to evaluate the real-world disease burden and describe the natural history of ISM by comparing patients with ISM treated at Mayo Clinic to a propensity-matched control cohort. In addition to standard methods using clinical codes for extraction of pertinent data from the Mayo Clinic EHR, NLP methods were used to transform unstructured information from clinical notes for additional data and context.

## Methods

### Study design overview

This was a retrospective cohort study of patients diagnosed with ISM at a Mayo Clinic location between January 1, 2005, and June 30, 2022. Patients with ISM were identified with both structured EHR data extraction and the use of NLP to review unstructured clinical notes based on the inclusion and exclusion criteria described in Table E1 (in the Online Repository available at www.jaci-global.org). A control cohort was developed on the basis of a 10:1 propensity matching using demographic and clinical variables. Elements in the EHR characterizing disease burden and natural history were then extracted for both the ISM and control cohorts using the structured (eg, demographics, vitals, laboratory tests, diagnosis codes, medications, and procedures) and unstructured EHR data (eg, physician notes and procedural reports).

### Data source and extraction

This study involves the analysis of deidentified EHR from Mayo Clinic Platform data via the nference Analytics Platform. Data shown and reported in this study have been extracted from this environment using an established protocol for data extraction, aimed at preserving patient privacy. The data have been deidentified pursuant to an expert determination in accordance with the Health Insurance Portability and Accountability Act (HIPAA) Privacy Rule. Any data beyond what are reported in this study, including but not limited to the raw EHR data, cannot be shared or released because of the parameters of the expert determination to maintain the data deidentification. Contact corresponding authors for additional details regarding the nference nSights Analytics Platform.

**Fig 1:**
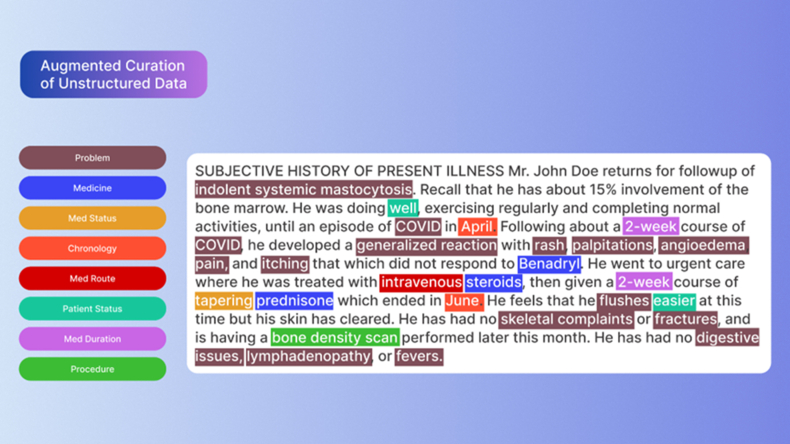
Example of nference augmented curation method. Each sentence of a clinical document undergoes entity recognition (highlighted terms); extracted entities undergo sentiment analysis within the context of the sentence by pretrained BERT models to determine presence of a procedure, medication, symptom, patient status, etc. In this text, “indolent systemic mastocytosis” would be labeled as “has disease,” whereas “digestive issues,” “lymphadenopathy,” and “fevers” would be labeled as “does not have disease.”

Patients were identified from the Mayo Clinic EHR data described above. These data include approximately 7.5 million patients from across all US-based Mayo Clinic sites. The Mayo Clinic EHR has records consistently available from 2005 onward, with some records dating back to 1990 or earlier. Mayo Clinic includes main campuses in Minnesota, Arizona, and Florida as well as a network of clinics, hospitals, and other health care facilities serving more than 60 communities in Minnesota, Wisconsin, and Iowa (Mayo Clinic Health System).

nference has developed an approach termed “augmented curation” that allows the scalable curation of medical records by leveraging neural networks that are trained using labeled clinical data that have been manually annotated by nference’s clinical scientists. Bidirectional Encoder Representations from Transformers (BERT) is a state-of-the-art machine learning technique for NLP that has been pretrained on a scientific corpus (3.3 billion words) and shows a state-of-the-art performance on a wide variety of tasks.^20^ nference has taken this pretrained BERT network and adapted it to multiple applications in the EHR, including entity extraction, classification, and relational tasks, through subsequent training steps to create novel models. These models allow for scalable curation of health records by leveraging neural networks that are trained using labelled data from Mayo Clinic following manual annotation by nference clinical scientists.^21^

nference’s augmented curation disease diagnosis model was used to classify the context of a mentioned disease, comorbidity, allergy, or symptom in patient clinical notes (Fig 1). This BERT-based model classifies mentions of disease or symptoms as “Yes” (confirmed), “Maybe” (possible), or “No” (ruled out). An “Other” label includes mentions such as family history or educational statements. Trained on approximately 31,000 sentences across 100+ diseases, the model achieves an F1 score of at least 80% for each entity and 85% for each label, with an average F1 score of 91% for the “Yes” label.^22^^,^^23^ The nference augmented curation medication administration model was used to determine instances of medication use in unstructured data, which may have been taken or prescribed outside of Mayo Clinic. Medication use is classified as “Received-Current,” “Received-Past,” “Timing Unclear,” “Not Received,” or “Other.” Validated on approximately 2000 sentences, the model achieves an average F1-score of 91% for the “Received-Current” label.

### Study population

This retrospective cohort study consisted of subjects 18 years or older (at diagnosis) who had been diagnosed with ISM at the Mayo Clinic from January 1, 2005, to June 30, 2022, and met all other key inclusion criteria and none of the exclusion criteria (Table E1). A control cohort was propensity score matched 10:1 using the following characteristics:

• Race
• Ethnicity
• Sex
• Age at index
• Quan-Charlson comorbidity index score
• Body mass index at index
• Smoking status

For the ISM cohort, in addition to having an ISM diagnosis in the 2005-2022 *index date* window, at least 1 diagnosis was also required in the October 2017-June 2022 *identification* window. Patients were initially diagnosed using *International Classification of Diseases, Tenth Revision* codes from a previously developed algorithm. In addition, NLP supplemented these codes, particularly during the period before 2017. A second encounter in the identification window was required because the institution of a diagnosis code in *International Classification of Diseases, Tenth Revision* specifically for “systemic mastocytosis” (D47.02) did not occur until October 1, 2017. Therefore, the identification window was implemented to validate pre-October 2017 ISM diagnoses.

**Fig 2:**
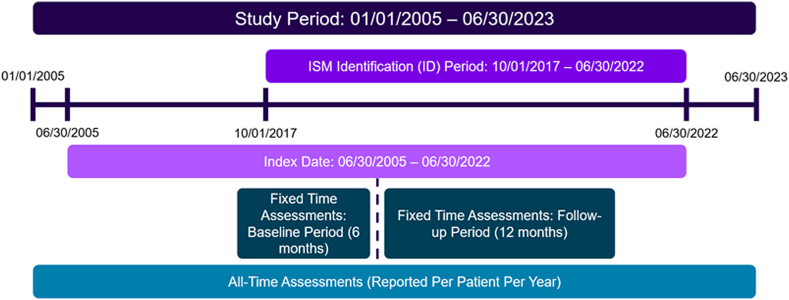
Study design timeline.

For the control cohort, the index date was defined as the date of the first encounter after 2 previous encounters during the study period. All subjects in both cohorts were required to have health records for 18 months. Baseline characteristics were assessed using data from the 6 months before the index date (*baseline* period). Outcomes were evaluated for 12 months following the index date (*follow-up* period) to better assess disease burden directly around time of diagnosis. Where applicable, some outcomes were evaluated beyond 12 months (*all-time* period) to look more broadly from a natural history perspective. The full study period encompassed 2005-2023. A study timeline schematic is provided in Fig 2.

### Study outcomes

During the 6-month baseline period, demographic characteristics and clinical variables, such as comorbid conditions and symptoms, were quantified and health care resource use for SM workup and diagnosis was evaluated. In the follow-up period after the index date, a longitudinal assessment of symptoms and resource use was conducted. Resources of interest included visits for outpatient, emergency department, inpatient, and critical care services, as well as medication use. Codes and nomenclature used to identify clinical and resource variables are presented in Table E2 (in Online Repository available at www.jaci-global.org).

### Data analysis

All study variables, including baseline and follow-up outcome measures, were analyzed descriptively, with numbers and percentages provided for categorical variables. Means, medians, SDs, and quartiles were provided for continuous variables. Descriptive techniques that account for length of observation time (eg, per-patient per-month amounts) were used where appropriate. Baseline characteristics and outcome measures were compared using appropriate statistical tests (eg, *t* test, Wilcoxon rank-sum, and chi-square test), based on the distribution of the measure.

## Results

### Patient identification and baseline characteristics

**Supplementary Fig E1:**
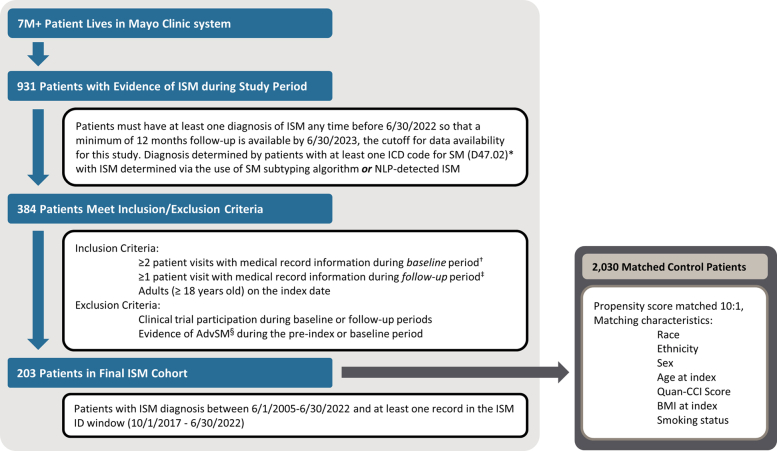


Of the more than 7 million patient-lives in the Mayo Clinic system, 384 patients with ISM were identified in the 2005-2022 index date window. Of these, 203 patients were eligible for the analysis based on the additional availability of 1 or more records in the 2017-2022 identification window. These patients with ISM were propensity-matched to 2030 control patients without ISM (see Fig E1 in this article’s Online Repository at www.jaci-global.org).

The mean age of the ISM cohort was 51.4 ± 15.7 years; 67% were female. Approximately 94% of the overall cohort was White. The average length of follow-up was 4.4 years. Additional demographic and cohort characteristics are presented in Table I.

**Table I:** Baseline demographic and clinical characteristics

Characteristic	ISM Cohort (N = 203)	Control cohort (N = 2030)
Age (y)		
Mean ± SD	51.4 ± 15.7	51.9 ± 16.3
18-44, n (%)	71 (35.0)	685 (33.8)
45-64, n (%)	86 (42.4)	833 (41.0)
65+, n (%)	46 (22.6)	512 (25.2)
Sex: female, n (%)	135 (66.5)	1372 (67.6)
Race and ethnicity, n (%)		
White or Caucasian	188 (92.6)	1923 (94.7)
Other race∗	15 (7.4)	107 (5.3)
Hispanic or Latino	10 (4.9)	84 (4.1)
Never smoker, n (%)	135 (66.5)	1379 (67.9)
Quan-CCI score, n (%)		
0	102 (50.2)	1046 (51.5)
1-4	84 (41.4)	793 (39.1)
5+	17 (8.4)	191 (9.4)
Year of index / ISM diagnosis, n (%)		
2005-2016	40 (19.7)	455 (22.4)
2017	16 (7.9)	182 (9.0)
2018	38 (18.7)	368 (18.1)
2019	33 (16.3)	294 (14.5)
2020	21 (10.3)	223 (11.0)
2021	35 (17.2)	328 (16.2)
2022	20 (9.9)	180 (8.9)
Distinct comorbidities per patient, n (%)		
0	54 (26.6)	1292 (63.7)
1	50 (24.6)	323 (15.9)
2-3	75 (37.0)	321 (15.8)
4-8	24 (11.8)	94 (4.6)
Distinct medication classes per patient, n (%)		
0	64 (31.5)	1229 (60.5)
1	21 (10.3)	241 (11.9)
2-3	46 (22.7)	339 (16.7)
4-9+	72 (35.5)	221 (10.9)
Biomarker prevalence, n (%)		
Elevated serum tryptase levels	23 (11.3)	0 (0.0)
Symptom prevalence, n (%)		
Fatigue	93 (45.8)	522 (25.7)
Nausea	84 (41.4)	433 (21.3)
Diarrhea	80 (39.4)	305 (15.0)
Muscle and/or joint pain	79 (38.9)	471 (23.2)
Anxiety and/or depression	74 (36.5)	486 (23.9)
Allergic reactions	72 (35.5)	285 (14.0)
Dyspnea	69 (34.0)	366 (18.0)
Compromised bone	69 (34.0)	322 (15.9)
Pruritus	68 (33.5)	186 (9.2)
Vomiting	62 (30.5)	337 (16.6)
Urticaria	57 (28.1)	105 (5.2)
Anaphylaxis	46 (22.7)	57 (2.8)
Abdominal pain and/or cramping	36 (17.7)	138 (6.8)
Weight loss	35 (17.2)	284 (14.0)
Hypotension	32 (15.8)	106 (5.2)
Syncope	32 (15.8)	71 (3.5)
Bloating	23 (11.3)	89 (4.4)
Lymphadenopathy	21 (10.3)	135 (6.7)
Flushing	19 (9.4)	12 (0.6)
Angioedema	18 (8.9)	8 (0.4)
Splenomegaly	11 (5.4)	45 (2.2)
Malabsorption	7 (3.4)	9 (0.4)
Peptic ulcer disease	7 (3.4)	35 (1.7)
GI bleeding	4 (2.0)	34 (1.7)
Hepatomegaly	3 (1.5)	17 (0.8)

*CCI*, Charlson comorbidity index; *GI*, gastrointestinal.

∗ Other includes “Asian or Asian American,” “Black or African American,” “Native American or Pacific Islander,” “Choose not to disclose,” or “Other.”

At baseline, patients with ISM had a higher prevalence of numerous symptoms and a higher mean number of distinct symptoms per patient than controls: 7.4 versus 4.8. Many symptoms were present in 30% or more of ISM patients with fatigue, nausea, diarrhea, muscle and/or joint pain, and anxiety and/or depression as the 5 most common. Similarly, baseline medication use was substantially higher in patients with ISM: 35% of patients with ISM had prescriptions for 4 to 9+ distinct medication classes compared with 11% for controls; conversely, only 32% of patients with ISM had no prescriptions within medication classes of interest compared with more than 60% for controls. Finally, a greater proportion of patients with ISM had at least 1 (up to 8) reported comorbidity (73%) compared with controls (36%).

### ISM workup and diagnosis

Access to EHR data that can supplement the information found in claims alone provides an opportunity to understand the real-world diagnostic workup that patients receive for ISM, including biopsies and genetic testing. Biopsies are a common medical procedure performed for tissue examination in those with suspected ISM. Biopsies of bone marrow, gastrointestinal tissues, and skin were conducted on more than 89% of patients with ISM, which was significantly greater (*P* < .0001; chi-square test) than for control subjects (<28% for the 3 biopsy types). For both the ISM and control cohorts, lymph node, liver, and lung biopsies were performed in less than 17% of patients (Table I).

Current standard of care for ISM diagnosis also includes genetic testing. However, given the extensive study period of this analysis, shifts have occurred in the availability and sensitivity of KIT testing over that time period. Of the 203 patients with ISM in whom genetic testing was performed, 98% (138 of 141) had testing for KIT mutation(s), with KIT detected in 54% (75 of 138) of patients; the average year of first KIT test completion was 2017, with increasing prevalence in following years. This detection rate is not representative of current practice for the reasons mentioned above.^24^^,^^25^

### Comorbidity and symptom burden

**Fig 3:**
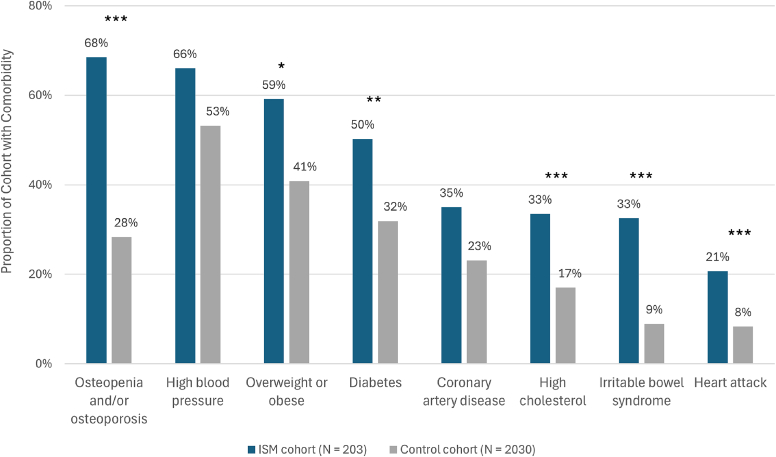
Proportion of cohort with comorbid conditions. Comorbidities were determined by both structured diagnosis codes and/or unstructured mentions in clinical notes via augmented curation during all-time follow-up. The *P* values of the ratio of patients with comorbidities (ISM:Control) were calculated by the chi-square test. ∗*P* < .05; ∗∗*P* < .01; ∗∗∗*P* < .001. All other evaluated comorbidities occurred at a frequency less than 5% in both cohorts.

Patients with ISM were demonstrated to have significantly higher rates of many comorbid conditions compared with controls (Fig 3). All-time rates of compromised bone (osteopenia, osteoporosis) was present in 68% of patients with ISM compared with 28% in controls (*P* < .0001; chi-square test). Other notable comorbidities with significantly higher prevalence (all time) in patients with ISM were obesity (59% vs 41%), diabetes (50% vs 32%), and high cholesterol (33% vs 17%; *P* < .05 for obesity, diabetes, and cholesterol comorbidities; chi-square test). Overall, 99% of patients in the ISM cohort had at least 1 reported comorbidity compared with 78% of patients in the control cohort (*P* < .0001; chi-square test). In addition, there were more comorbidities per patient in the ISM cohort than in controls (4.7 ± 2.2 vs 2.2 ± 1.8; *P* < .0001; unpaired 2-tailed Student *t* test).

**Fig 4:**
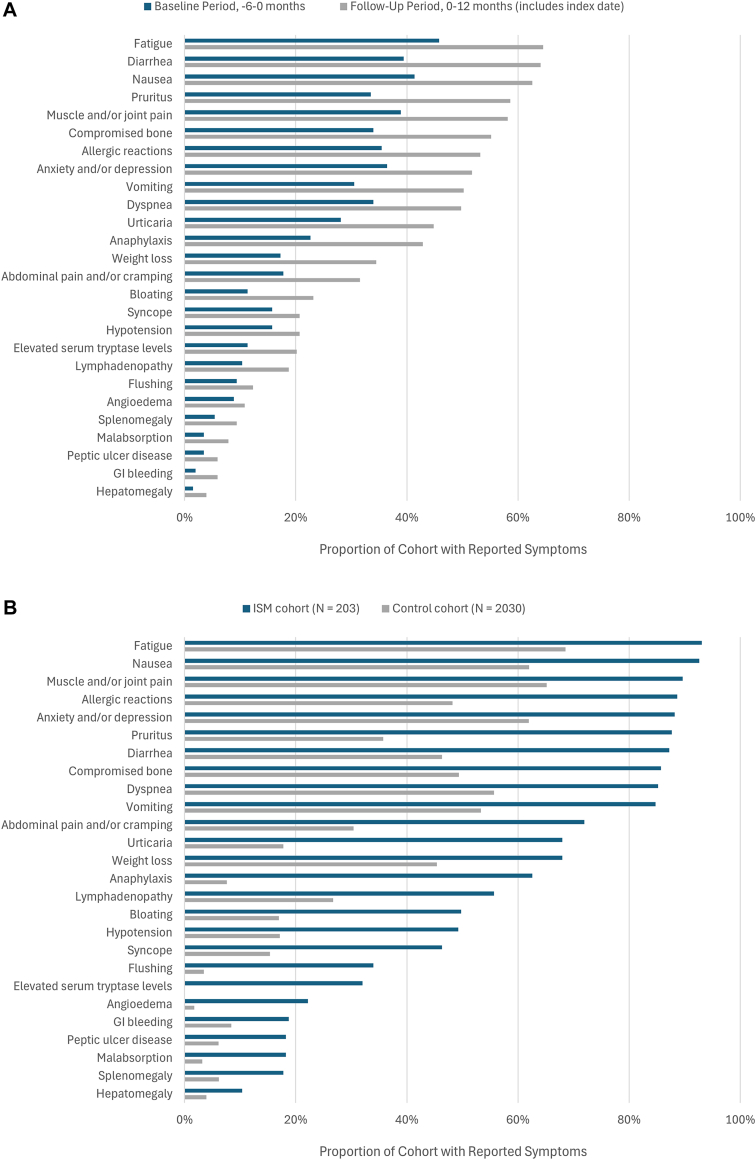
Symptom prevalence. **A,** Individual ISM symptoms at baseline and 12-month follow-up. **B,** All-time symptom prevalence in ISM and control cohorts. *GI*, Gastrointestinal. Symptoms were determined by both structured diagnosis codes and/or unstructured mentions in clinical notes via augmented curation.

ISM is characterized by numerous, heterogeneous symptoms. This was borne out by this analysis of EHR data. During both the baseline and follow-up periods, individuals with ISM were shown to experience numerous general symptoms (eg, anxiety, depression, and fatigue), gastrointestinal symptoms (eg, abdominal pain and diarrhea), dermatological symptoms (eg, pruritus and urticaria), systemic symptoms (eg, compromised bone and syncope), mediator-related symptoms (eg, anaphylaxis and allergic reactions), and biomarkers related to mast cell activation (eg, elevated serum tryptase levels) (Fig 4, *A*).

During the 6-month baseline period, patients with ISM most commonly experienced dermatologic symptoms (angioedema, flushing, pruritus, and urticaria [55%]), fatigue (46%), nausea (41%), and diarrhea (39%). Other symptoms of particular concern, given their high burden (or potentially life-threatening nature), included compromised bone (34%) and anaphylaxis (23%). The prevalence of these, and all remaining symptoms, increased after the index date during the 12-month follow-up period. Overall, 99% of patients in the ISM cohort had at least 1 reported symptom and had a greater mean number of distinct symptoms in the follow-up period (9.3 symptoms) than in the baseline period (7.4 symptoms).

The all-time symptom prevalence, considering available data greater than 12 months postindex date, for both ISM and controls was evaluated (Fig 4, *B*). The mean number of distinct symptoms per patient (ISM vs control) was 15.7 ± 4.3 and 7.8 ± 4.2 (*P* < .0001; unpaired 2-tailed *t* test). The symptom profile in patients with ISM was similar to the baseline and 12-month follow-up periods in that there was a high prevalence of many gastrointestinal, systemic, and general symptoms. Notably, 89% of patients with ISM had allergic reactions and 63% had anaphylaxis.

**Fig 5:**
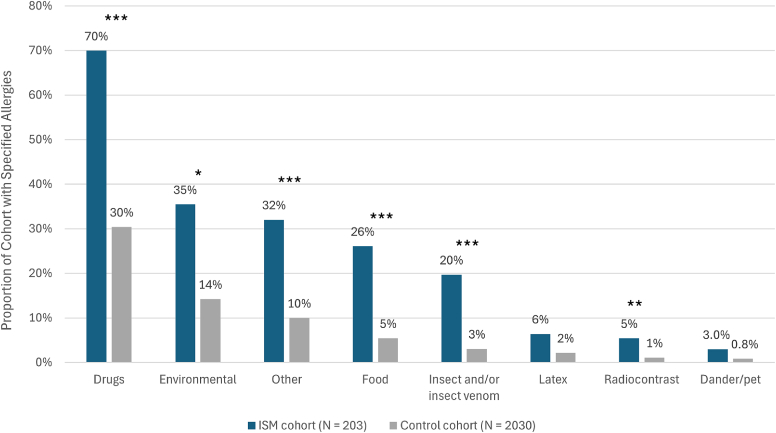
Allergies present in ISM and control cohorts. Allergies were determined by both structured diagnosis codes and/or unstructured mentions in clinical notes via augmented curation during all-time follow-up. The *P* values of the ratio of patients with allergies (ISM:Control) were calculated by the chi-square test. ∗*P* < .05; ∗∗*P* < .01; ∗∗∗*P* < .001.

The EHR data allowed a more detailed inspection of allergic reactions and categorization by type of trigger. Patients with ISM were significantly more likely to have allergies to medications (70% vs 30%) and environmental triggers (including animals and pollen; 35% vs 14%) than controls (Fig 5). In this analysis, 84% of patients in the ISM cohort had at least 1 reported allergy compared with 42% of patients in the control cohort (*P* < .0001; chi-square test).

### Health care resource utilization

**Fig 6:**
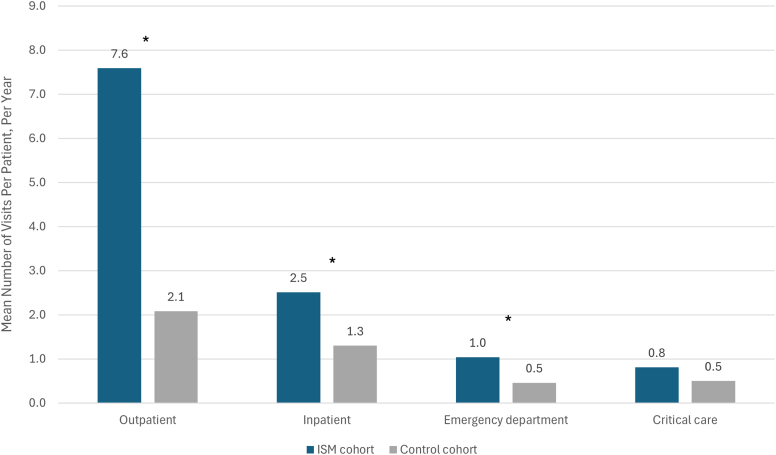
Health care resource utilization: mean visits per patient per year. The frequency of resource use was described on a per patient-year basis among patients with at least 1 visit. For each individual patient, the total number of visits was divided by the number of months or years from the index date to the last recorded contact date. The *P* values of means were calculated by unpaired 2-tailed Student *t* test or the unpaired 2-tailed Mann-Whitney *U* test if nonparametric. ∗*P* < .001.

Given the potential clinical impact of ISM comorbidities and symptoms, it was hypothesized that patients with ISM may be high utilizers of different health care resources. Indeed, the analysis of the EHR data showed that patients with ISM had greater utilization of most types of health care resources (Fig 6). The mean annual number of visits for outpatient care, inpatient admissions, and emergency department visits were all statistically significantly higher for patients with ISM compared with controls (*P* < .001; unpaired, 2-tailed Student *t* test or unpaired 2-tailed Mann-Whitney *U* test if nonparametric). Critical care visits were not significantly different between groups. Annual outpatient visit frequency demonstrated the greatest difference (7.6 visits per patient vs 2.1 visits).

**Fig 7:**
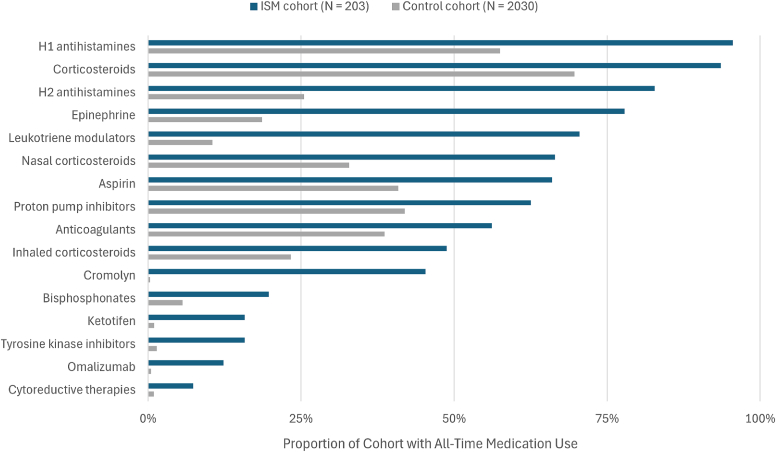
Health care resource utilization: medication use. Medication data were extracted from structured medication orders/administrations and/or an augmented curation medication-administered model on the unstructured clinical text during all-time follow-up.

Patients with ISM also are high utilizers of many types of medications (Fig 7). More than 78% of patients with ISM use or have used H1 or H2 antihistamines, corticosteroids, or epinephrine. In addition to these medications, many others were used by a significantly higher proportion of patients with ISM than controls including leukotriene modulators, aspirin, proton pump inhibitors, anticoagulants, and cromolyn. On average, patients with ISM take more classes of medication than do controls (14.7 vs 5.8).

The Mayo Clinic EHR data also allow insights into the temporal aspects of medication use relative to an ISM diagnosis. For example, proton pump inhibitors are one of the first medications ordered or mentioned before a patient having an ISM diagnosis (∼4-6 years before diagnosis). In contrast, omalizumab and ketotifen are ordered or mentioned most closely to the actual diagnosis of ISM (∼6-18 months before diagnosis).

## Discussion

This study of patients with ISM within Mayo Clinic used a unique NLP algorithm applied to unstructured clinical text to augment structured claims and laboratory data. Given that the *International Classification of Diseases, Tenth Revision* code for systemic mastocytosis (D47.02) did not become available until 2017, using NLP allowed more patients with ISM to be identified than would have been identified by diagnosis code alone. Relevant comorbidities and allergies not captured in claims data can also be identified with NLP to better assess disease burden.^26^^,^^27^ The inclusion of unstructured data elements is essential to providing a comprehensive view of the real-world patient experience of patients with ISM and a more comprehensive understanding of the natural history of this chronic disease.

Because of the unique nature of Mayo Clinic, including the role it may play providing specialty care for patients both regionally and locally, the type and quality of care received by patients in this system may differ in meaningful ways from that patients receive in other, less specialized centers. For instance, because of the urgent nature of many emergency department visits, we expect the reported frequency of that resource to be underestimated given the likelihood that patients may occasionally seek emergency care outside Mayo Clinic. In addition, because of the specialized nature of care at the Mayo Clinic, patients referred for treatment may not be representative of the overall ISM population and their experience with the disease may not be generalizable to a broader ISM population. For example, their disease may be of greater severity or complexity, leading to the referral and subsequent treatments.

Other results may be more related to study design than setting. For example, observed differences in medication use between ISM and controls may be overstated because the analysis did not capture “over-the-counter” products. Furthermore, a lengthy study period, over which time practice patterns and technology may evolve, could influence our observations and require additional context, with genetic testing providing a good example. It comes as no surprise then that most patients in this study had received a diagnostic biopsy of some type. Genetic testing is only more recently seen as standard of care in treating patients with ISM and so it is not unexpected that prevalence of KIT testing has increased since 2017. Relatively low rates of KIT D816V testing and positivity, likely due to the primary use of next-generation sequencing testing methodologies as the available technology at the time of most observed testing, highlight the importance of adopting new high-sensitivity testing for accurate diagnosis.

Consistent with previous research into the burden of ISM, the patients in this study experienced a high burden of heterogeneous and unpredictable symptoms, including gastrointestinal, skin, neurocognitive, systemic, and other symptoms. Notably, this study demonstrates that patients not only experience a broad range of symptoms across the disease state but many patients experience numerous symptoms, with an average of 9.3 symptoms in the follow-up period. In addition, various allergies were present in most patients and 23% of patients experienced more severe anaphylaxis in the 12-month follow-up period. Of note, 63% of patients showed evidence of at least 1 case of anaphylaxis across the full time period of this study. This result, and others that were evaluated over a long time period in this study using “all-time” rates, may therefore be higher than reported in published literature to date.

Compared with matched controls, patients with ISM were more likely to have at least 1 comorbid condition, had on average twice as many comorbid conditions, and were more likely than patients without ISM to experience obesity, diabetes, and high blood pressure. Patients with ISM are often unable to engage in normal physical activity, report higher levels of stress and anxiety, and due to allergic reactions and possible anaphylaxis triggers may have restricted diets.^15^^,^^17^ All these factors may contribute to the higher rates of comorbidity observed in this patient population and, regardless of the etiology of each condition, make these patients more complicated to manage.^28^^,^^29^

Not surprisingly, given the variety and number of symptoms, patients had a high polypharmacy burden, taking an average of 14.7 different classes of medication during the study period, many of them symptom-directed. Although symptoms driven by mast cell mediators (eg, allergic reaction and anaphylaxis) and mast cell accumulation (eg, elevated tryptase and associated effects) may be managed and even resolved with some of these symptomatic treatments, other effects of disease are less transient and require actual disease modification to be fully addressed. The bone loss that may result from high levels of modulatory bone cytokines seen in ISM can have lasting and meaningful effects on patients’ risk for fractures and other negative outcomes associated with osteoporosis and osteopenia. The symptom burden demonstrated by these patients is consistent with previous research in ISM and has been correlated elsewhere with significant decreases in both health-related and broader quality of life, including well-being and patients’ ability to work.^17^^,^^30^

There are several notable strengths of this study. Because ISM is a chronic disease and some studies have shown that it may take 6 to 9 years for patients to receive a diagnosis,^15^^,^^30^ longitudinal data are critical to fully understand the natural history of the disease. A major strength of this analysis is the ability to follow patients for an average of 53 months postdiagnosis, allowing for a more comprehensive understanding of the symptom and health care resource use burden associated with ISM. Leveraging NLP to analyze unstructured data elements further strengthened this study. However, this study uses data only from Mayo Clinic. Many patients do not use Mayo Clinic for all their health care (eg, they may receive specialty care there but may seek emergency care at a local center). In addition, because Mayo Clinic serves as a source of specialty care for many patients, the results of this study may not be representative outside of this system. Patient demographic homogeneity may also limit the broader generalizability of these results. Further research on the natural history of ISM in non-White populations is needed.

Although patients treated within the Mayo Clinic may differ from the overall patient population in meaningful ways, these data provide insight into the treatment gaps and disease burden for all patients with ISM, as demonstrated by their consistency with previously published literature. A treatment paradigm focused merely on providing symptomatic relief may fail to adequately address long-term sequelae resulting from the broad-based mast cell proliferation and infiltration that characterize the disease. The elevated prevalence of bone involvement in Mayo Clinic patients with ISM may provide evidence for the importance of disease-modifying therapies, not just to treat symptoms of the disease but also to prevent long-term damage.

**Clinical implications**

Patients with ISM experience high symptom burden, comorbidities, and health care utilization, underscoring the need for earlier diagnosis and comprehensive, multidisciplinary management strategies.

## Disclosure statement

This research was funded by Blueprint Medicines Corporation.

Disclosure of potential conflict of interest: T. Pongdee receives research funding from Blueprint Medicines Corporation and Sanofi. A. Smither and P. Krishnappa are employees of nference. P. Singh is an employee of nference and owns nference stock. K. Carlson was an employee of nference at the time of the study. D. Powell, T. Weis, R. Scherber, T. Green, and E. Sullivan are employees of Blueprint Medicines Corporation. S. Duff is an employee of Veritas Health Economics Consulting and consultant for Blueprint Medicines Corporation.

## Supplementary data

Supplementary Tables E1-E2 and Fig E1
