# Supplementary material for: Assessing real-world natural history of indolent systemic mastocytosis: A retrospective matched cohort study
Source: J Allergy Clin Immunol Glob. 2026 Apr 13;5(4):100701. doi: 10.1016/j.jacig.2026.100701 (PMC13157138; doi:10.1016/j.jacig.2026.100701)
Supplement: Supplementary Tables E1-E2 and Fig E1 [file mmc1.docx]

**Supplemental Tables and Figures**

**Supplemental Table I.** Inclusion / exclusion criteria for ISM cohort*

| **Inclusion Criteria** | **Exclusion Criteria** |
| --- | --- |
| Diagnosis determined by patients with at least one ICD code for SM (D47.02)^†^ with ISM determined via use of SM subtyping algorithm or NLP-detected ISM | Clinical trial participation during baseline or follow-up periods |
| ≥2 patient visits with medical record information during the *baseline* period | Evidence of AdvSM during the pre-index or baseline period |
| ≥1 patient visit with medical record information during the *follow-up* period |  |
| ≥1 patient visit with medical record information during the 10/1/17 – 6/30/22 *identification* period |  |
| Adults (≥18 years old) on the index date |  |

*AdvSM*, advanced systemic mastocytosis; *BMI*, body mass index; *CCI*, Charlson Comorbidity Index; *ICD*, International Classification of Diseases; *ISM*, indolent systemic mastocytosis; *SM*, systemic mastocytosis.

*Control cohort developed using propensity score matching on: race, ethnicity, sex, age at index, Quan-CCI score, BMI at index, smoking status

^†^This diagnosis code was added to the International Classification of Disease, Tenth Revision (ICD-10) coding system in 2017.

**Supplemental Table II.** Descriptions, codes, and synonyms for disease, symptoms, comorbidity, and resource variables

| **ICD-10 Codes** | **Description** | **Synonyms** |  |
| --- | --- | --- | --- |
| **ISM Diagnosis** |  |  |  |
| D47.02 | Systemic mastocytosis | systemic mastocytosis, systemic mast cell disease, mast cell disease, systemic mastocytoses, smcd, systemic tissue mast cell disease |  |
| D47.09 | Other mast cell neoplasms of uncertain behavior | mast cell neoplasms, mast cell proliferative disorders, mcts, mast cell neoplasia, mast cell tumors |  |
| C96.20 | Malignant mast cell neoplasm, unspecified | malignant mast cell neoplasm |  |
| C96.22 | Mast cell sarcoma | mast cell sarcoma, malignant mast cell tumor, malignant mastocytoma, mcs |  |
| C96.29 | Other malignant mast cell neoplasms | malignant mast cell neoplasm |  |
| **Coexisting/comorbid conditions; clinical relevant events and symptoms** | **Description** | **Diagnosis Codes (parent)** | **Synonyms** |
| **Allergies^1^** | Food | **ICD-9**  477.1, 995.6*, V15.01, V15.02, V15.03, V15.04, V15.05  **ICD-10**  J30.5, T78.0*, Z91.01* | almond, corn, egg, fish, food, gluten, milk, nut, peanut, shellfish, soy, tree nut, wheat, food anaphylaxis^ |
|  | Dander/pet | **ICD-9**  477.2  **ICD-10**  J30.81 | animals, cats, dogs, horses |
|  | Insect and/or insect venom | **ICD-9**  E905.3, E905.4, E905.5, V15.06  **ICD-10**  T63.4*, Z91.03* | bee sting^, venom, bites and stings^ |
|  | Environmental (including pollen and plants) | **ICD-9**  477.0  **ICD-10**  J30.1, J30.2 | pollen, dust mite, environmental, fungal, mold, perennial, ragweed, seasonal |
|  | Drugs | **ICD-9**  995.27, V14.*  **ICD-10**  T88.6*, Z88.* | amiodarone, amlodipine, amitriptyline, amoxicillin, aspirin, carbapenem, cefoxitin, chlorhexidine, citalopram, ciprofloxacin, colchicine, drug, enoxaparin, erythromycin, fentanyl, heparin, hyaluronic acid, ketoconazole, lamotrigine, lidocaine, metronidazole, midazolam, minoxidil, morphine, naproxen, prednisone, ranitidine, simvastatin, sulfamethoxazole, sulfa, tamoxifen, theophylline, trimethoprim, vancomycin |
|  | Latex | **ICD-9**  V15.07  **ICD-10**  Z91.040 | latex |
|  | Radiocontrast | **ICD-9**  V15.08  **ICD-10**  Z91.041 | iodine contrast, contrast media |
|  | Other | **ICD-9**  477.8, 477.9, 995.0, 995.3, 995.4*, V15.09  **ICD-10**  J30.89, J30.9, T78.2*, T80.5*, Z91.048, Z91.09 | tape, nickel, sun, cold, transfusion reactions |
| **Diabetes** |  | **ICD-9**  249.*, 250.*  **ICD-10**  E08.*, E09.*, E10.*, E11.*, E13.* | diabetes |
| **Obesity or overweight** |  | **ICD-9**  278.0*, V85.2*, V85.3*, V85.4*  **ICD-10**  E66.*, Z68.25, Z68.26, Z68.27, Z68.28, Z68.29 Z68.3*, Z68.4* | obesity, morbid obesity, overweight |
| **Osteopenia or osteoporosis** | | **ICD-9**  733.0*, 733.90  **ICD-10**  M80.*, M81.*, M85.8* | osteoporosis, osteopenia |
| **Myelodysplastic syndromes** |  | **ICD-9**  238.72, 238.73, 238.74, 238.75  **ICD-10**  D46.* | refractory anemia, sideroblastic anemia, myelodysplastic syndrome, 5q syndrome |
| **Chronic myelomonocytic leukemia (CMML)** |  | **ICD-9**  206.1*  **ICD-10**  C93.1* | chronic myelomonocytic leukemia |
| **Acute myeloblastic leukemia (AML)** |  | **ICD-9**  205.0*  **ICD-10**  C92.0*, C92.6*, C92.A* | acute myeloblastic leukemia |
| **Coronary artery disease (Atherosclerosis)** |  | **ICD-9**  414.0*, 414.2*, 414.3*, 414.4*  **ICD-10**  I25.1*, I25.7*, I25.81*, I25.83, I25.84 | atherosclerosis, atherosclerotic disease, coronary artery disease |
| **Hypertension** |  | **ICD-9**  401.*, 405.*  **ICD-10**  I10.*, I15.*, I1A.* | hypertension, high blood pressure |
| **High cholesterol** |  | **ICD-9**  272.0  **ICD-10**  E78.0* | high cholesterol, hypercholesterolemia |
| **Myocardial infarction** |  | **ICD-9**  410.*, 412  **ICD-10**  I21.*, I22.* | myocardial infarction, non stemi |
| **Irritable bowel syndrome** | | **ICD-9**  564.1  **ICD-10**  K58.* | irritable bowel syndrome |
| **Hereditary alpha tryptasemia (HaT)** | | **ICD-9**  N/A  **ICD-10**  D89.44 | hereditary alpha tryptasemia |
| **General symptoms** | Pruritus | **ICD-9**  698.*  **ICD-10**  L29.* | pruritus |
|  | Flushing | **ICD-9**  782.62  **ICD-10**  R23.2 | flushing |
|  | Urticaria | **ICD-9**  708.*  **ICD-10**  L50.* | urticaria |
|  | Fatigue | **ICD-9**  780.71, 780.79  **ICD-10**  G93.32, R53.82, R53.83 | chronic fatigue syndrome, fatigue, tired |
|  | Muscle and/or joint pain | **ICD-9**  719.4*, 729.1, 729.5  **ICD-10**  M25.5*, M79.1*, M79.6*, M79.7 | arthralgia, myalgia, fibromyalgia, limb pain, toe pain, hand pain, wrist pain, ankle pain, finger pain |
|  | Anxiety and/or depression | **ICD-9**  296.2*, 296.3*, 300.0*, 300.4  **ICD-10**  F32.*, F33.*, F34.1, F41.* | depression, depressive, anxiety, generalized anxiety disorder, panic, dysthymic disorder |
|  | Weight loss | **ICD-9**  783.21  **ICD-10**  R63.4 | weight loss |
| **GI symptoms** | Abdominal pain and/or cramping | **ICD-9**  789.0*  **ICD-10**  R10.0, R10.1*, R10.3* R10.84, R10.9 | abdominal cramps, gastric pain, upset stomach, stomach cramps, stomach pain |
|  | Bloating | **ICD-9**  787.3  **ICD-10**  R14.0, R14.1 | abdominal distention, bloating, gas bloat, gas pain |
|  | Diarrhea | **ICD-9**  564.1, 564.5, 787.91  **ICD-10**  K58.0, K58.9, K59.1, R19.7 | diarrhea, diarrhoea, irritable bowel syndrome |
|  | Nausea | **ICD-9**  787.01, 787.02  **ICD-10**  R11.0, R11.2 | nausea, nausea and vomiting |
|  | Malabsorption | **ICD-9**  579.8, 579.9  **ICD-10**  K90.4*, K90.82*, K90.89, K90.9 | malabsorption, short bowel syndrome |
|  | Vomiting | **ICD-9**  569.87, 787.01, 787.03, 787.04  **ICD-10**  R11.10, R11.11, R11.12, R11.13, R11.14, R11.2 | vomiting, nausea and vomiting |
|  | Gastrointestinal bleeding | **ICD-9**  578.0, 578.1, 578.9  **ICD-10**  K92.0, K92.1, K92.2 | hematemesis, melena, gastrointestinal hemorrhage |
|  | Peptic ulcer disease | **ICD-9**  531.*, 532.*, 533.*  **ICD-10**  K25.*, K26.*, K27.* | gastric ulcer, duodenal ulcer, peptic ulcer, peptic ulcer disease |
| **Mediator-related symptoms** | Anaphylaxis | **ICD-9**  995.0, 995.6*, 999.4*, V13.81  **ICD-10**  T78.0*, T78.2*, T80.5*, T88.6*, Z87.892 | anaphylaxis |
|  | Allergic reactions | **ICD-9**  477.0, 477.1, 477.2, 477.8, 477.9*, 558.3, 692.4, 692.5, 692.6, 692.81, 692.83, 692.84, 692.89, 692.9, 989.5, 995.27, 995.3, E905.3, E905.4, E905.5, V14.*, V15.0*  **ICD-10**  J30.1, J30.2, J30.5, J30.8*, J30.9, K52.29, L23.*, T63.4*, T78.40*, T78.49*, Z88.*, Z91.0* | allergic rhinitis, allergy, allergies, hypersensitivity, allergic, allergic contact dermatitis, gastrointestinal allergies, allergic colitis, allergic gastroenteritis |
| **Systemic symptoms** | Dyspnea | **ICD-9**  786.02, 786.05  **ICD-10**  R06.00, R06.01, R06.02, R06.09 | dyspnea, increased shortness of breath, orthopnea |
|  | Lymphadenopathy | **ICD-9**  785.6  **ICD-10**  R59.* | lymphadenopathy, swollen lymph nodes |
|  | Splenomegaly | **ICD-9**  789.2  **ICD-10**  R16.1, R16.2 | splenomegaly, hepatomegaly and splenomegaly |
|  | Syncope | **ICD-9**  780.2  **ICD-10**  R55 | syncope |
|  | Hypotension | **ICD-9**  458.*  **ICD-10**  I95.* | hypotension |
|  | Hepatomegaly | **ICD-9**  789.1  **ICD-10**  R16.0, R16.2 | hepatomegaly, hepatomegaly and splenomegaly |
|  | Angioedema | **ICD-9**  995.1  **ICD-10**  T78.3* | angioedema |
|  | Compromised bone | **ICD-9**  268.2, 588.00, 715.*, 731.2, 731.8, 732.7, 732.8, 732.9, 733.0*, 733.1*, 733.4*, 733.90, 733.93, 733.94, 733.95, 733.96, 733.97, 733.98, 733.99, 756.5*, 996.44, 996.45, V13.51, V13.52  **ICD-10**  M15.*, M16.*, M17.*, M18.*, M19.*, M48.4*, M48.5*, M80.*, M81.*, M83.0, M83.1, M83.3, M83.5, M83.8, M84.3*, M84.4*, M84.5*, M84.6*, M85.8*, M87.*, M89.4*, M89.5*, M90.*, M93.2*, M93.8*, M93.9*, M97.*, N25.0*, Q78.*, T84.05*, Z87.31* | osteopenia, osteoarthritis, osteomalacia, osteoporosis, osteonecrosis, exostosis, osteolysis, osteochondropathy, osteochondritis dissecans, compression fractures, vertebral collapse, pathologic fractures, stress fracture of the metatarsals, stress fracture of shaft of femur, osteoporotic fractures, stress fractures, occult fractures, hypertrophic osteoarthropathy, periprosthetic fracture, renal osteodystrophy, osteogenesis imperfecta, polyostotic fibrous dysplasia, osteopetrosis, camurati engelmann disease, enchondromatosis, metaphyseal dysplasia, familial exostoses, multiple epiphyseal dysplasia, skeletal dysplasia |
| **Mast cell activation biomarkers** | Elevated serum tryptase levels | Assess by lab values only | |
| **Drug Class** | **Drug Name (Brand Name(s))** | | |
| **H1 antihistamines** | Acrivastine (Semprex)  Brompheniramine (Dimetapp, bromphen)  Carbinoxamine (Karbinal)  Cetirizine (Zyrtec, Aller-Tec)  Chlorpheniramine (Chlor-Trimeton, Aller-Chlor)  Clemastine (Tavist)  Cyproheptadine (Periactin)  Desloratadine (Clarinex)  Dexchlorpheniramine (Polaramine)  Diphenhydramine (Benadryl, Nytol, Sominex)  Fexofenadine (Allegra)  Hydroxyzine (Atarax, Vistaril)  Levocetirizine (Xyzal)  Loratadine (Claritin, Alavert)  Promethazine (Phenergan, Promacot)  Rupatadine (Rupafin)  Triprolidine (Histex) | | |
| **Anticoagulants** | Warfarin (Coumadin, Jantoven)  Rivaroxaban (Xarelto)  Apixaban (Eliquis)  Fondaparinux (Arixtra)  Edoxaban (Savaysa)  Betrixaban (Bevyxxa)  Enoxaparin (Lovenox)  Dalteparin (Fragmin)  Tinzaparin (Innohep)  Danaparoid (Orgaran)  Heparin  Dabigatran (Pradaxa)  Angiomax (Bivalirudin)  Desirudin (Iprivask) | | |
| **Aspirin** | Aspirin | | |
| **Bisphosphonates** | Alendronate (Fosamax)  Clodronate (Bonefos)  Etidronate (Didronel)  Ibandronate (Boniva)  Neridronate (Nerixia)  Pamidronate (Aredia)  Risedronate (Actonel)  Tiludronate (Skelid)  Zoledronic acid (Reclast, Aclasta) | | |
| **Oral corticosteroid**  **Nasal corticosteroid**  **Inhaled**  **corticosteroid** | Azelastine^#^  Beclomethasone (Qvar^#/##^)  Betamethasone (Celestone)  Budesonide (Uceris, Entocort, Pulmicort^##^)  Ciclesonide^#^ (Omnaris, Zetonna)  Cortisone  Deflazacort (Emflaz)  Dexamethasone (Decadron)  Fludrocortisone  Flunisolide* (Aerobid)  Fluticasone (Flonase/Veramyst^#^, Flovent^##^)  Hydrocortisone  Methylprednisolone (Merdol)  Mometasone (Nasonex^#^, Asmanex^##^)  Prednisolone (Orapred, Prelone)  Prednisone (Deltasone, Rayos)  Triamcinolone (Kenalog, Nasocort^#^) | | |
| **Cromolyn (mast cell stabilizer)** | Cromolyn (Intal, Gastrocrom) | | |
| **Cytoreductive therapies** | Anagrelide (Agrylin)  Azacitidine (Vidaza)  Busulfan (Myleran)  Cladribine (Leustatin, Mavenclad)  Decitabine (Dacogen)  Hydroxyurea  Interferon-ɑ (Interferon alfa, Multiferon)  Ruxolitinib (Jakafi) | | |
| **Epinephrine injectors** | Adrenaclick  Auvi-Q  EpiPen  EpiPen Jr.  Symjepi | | |
| **H2 antihistamines** | Cimetidine (Tagamet)  Famotidine (Pepcid)  Nizatidine (Axid)  Ranitidine (Zantac)  Roxatidine (Roxatidine acetate hydrochloride) | | |
| **Ketotifen**  **(H1 antihistamine and mast cell stabilizer)** | Ketotifen (Zaditor) | | |
| **Leukotriene modulators** | Montelukast (Singulair)  Zafirlukast (Accolate)  Zileuton (Zyflo) | | |
| **Omalizumab** | Omalizumab (Xolair) | | |
| **Proton pump inhibitors** | Dexlansoprazole (Dexilant)  Esomeprazole (Nexium)  Lansoprazole (Prevacid)  Omeprazole (Prilosec)  Pantoprazole (Protonix)  Rabeprazole (Aciphex) | | |
| **Tyrosine kinase inhibitors** | Acalabrutinib (Calquence)  Afatinib (Gilotrif)  Alectinib (Alecensa)  Avapritinib (Ayvakit)  Axitinib (Inlyta)  Bosutinib (Bosulif)  Brigatinib (Alunbrig)  Cabozantinib (Cometriq, Cabometyx)  Campmatinib (Tabrecta)  Ceritinib (Zykadia)  Crizotinib (Xalkori)  Dacomitinib (Vizimpro)  Dasatinib (Sprycel)  Erdafitinib (Balversa)  Erlotinib (Tarceva)  Gefitinib (Iressa)  Ibrutinib (Imbruvica)  Imatinib (Gleevec)  Lapatinib (Tykerb)  Larotrectinib (Vitakvi)  Lenvatinib (Lenvima)  Lorlatinib (Lorbrena)  Midostaurin (Rydapt, Tauritmo)  Nilotinib (Tasigna)  Osimertinib (Tagrisso)  Pazopanib (Votrient)  Pemigatinib (Pemazyre)  Pexidartinib (Turalio)  Ponatinib (Iclusig)  Regorafenib (Stivarga)  Repretinib (Qinlock)  Sorafenib (Nexavar)  Selpercatinib (Retevmo)  Sunitinib (Sutent)  Tucatinib (Tukysa)  Trametinib (Mekinist)  Vandetanib (Caprelsa)  Zanubrutinib (Brukinsa) | | |

^1^Synonyms for allergies in clinical notes must have included “allergic to” before, or “allergy” or “allergies” after, the listed synonym text in order to be classified as an allergy to a substance, except where otherwise indicated (^).

^Allergy synonym searched for directly.

#, ##Represent nasal and inhaled corticosteroids, respectively, with the remainder as oral corticosteroids.

*Designates a wildcard character; any codes encompassed after the asterisk are included.

**203 Patients in Final ISM Cohort**

**931 Patients with Evidence of ISM during Study Period**

**384 Patients Meet Inclusion/Exclusion Criteria**

**7M+ Patient Lives in Mayo Clinic system**

Patients must have at least one diagnosis of ISM any time before 6/30/2022 so that a minimum of 12 months follow-up is available by 6/30/2023, the cutoff for data availability for this study. Diagnosis determined by patients with at least one ICD code for SM (D47.02)* with ISM determined via the use of SM subtyping algorithm ***or*** NLP-detected ISM

Inclusion Criteria:

≥2 patient visits with medical record information during *baseline* period^†^

≥1 patient visit with medical record information during *follow-up* period^‡^

Adults (≥ 18 years old) on the index date

Exclusion Criteria:

Clinical trial participation during baseline or follow-up periods

Evidence of AdvSM^§^ during the pre-index or baseline period

Patients with ISM diagnosis between 6/1/2005-6/30/2022 and at least one record in the ISM ID window (10/1/2017 - 6/30/2022)

**2,030 Matched Control Patients**

Propensity score matched 10:1,

Matching characteristics:

Race

Ethnicity

Sex

Age at index

Quan-CCI Score

BMI at index

Smoking status

**Supplemental FIG 1.** Patient cohort identification. Eligible patients were identified and propensity score matched with similar patients without ISM. *This diagnosis code was added to the International Classification of Disease, Tenth Revision (ICD-10) coding system in 2017. ^†^The 6-month period prior to the index data occurring any time between 6/30/2005-6/30/2022. ^‡^The 12-month period after the index date. ^§^Includes evidence of diagnostic coding for mast cell leukemia, mast cell neoplasms, or other hematologic neoplasms. *AdvSM*, advanced systemic mastocytosis; *BMI*, body mass index; *CCI*, Charlson Comorbidity Index; *ICD*, International Classification of Disease; *ISM*, indolent systemic mastocytosis.
